# Increase in circulating holotranscobalamin after oral administration of cyanocobalamin or hydroxocobalamin in healthy adults with low and normal cobalamin status

**DOI:** 10.1007/s00394-017-1553-5

**Published:** 2017-10-16

**Authors:** Eva Greibe, Namita Mahalle, Vijayshri Bhide, Christian W. Heegaard, Sadanand Naik, Ebba Nexo

**Affiliations:** 1 Department of Clinical Biochemistry and Institute of Clinical Medicine, Aarhus University Hospital, Aarhus, Denmark; 2 Department of Pathology, Deenanath Mangeshkar Hospital and Research Center, Pune, India; 3 Department of Molecular Biology and Genetics, Aarhus University, Aarhus, Denmark; 4 Clinical Biochemistry, Deenanath Mangeshkar Hospital and Research Center, Pune, India

**Keywords:** Vitamin B12, Cyanocobalamin, Hydroxocobalamin, Holotranscobalamin, CobaSorb test, Cobalamin absorption

## Abstract

**Purpose:**

To investigate the absorption of synthetic cyanocobalamin and natural occurring hydroxocobalamin in populations with low and normal cobalamin (vitamin B12) status.

**Methods:**

We included adults with low (*n* = 59) and normal (*n* = 42) cobalamin status and measured the change in serum holotranscobalamin (ΔholoTC) before and after 2 day administration of different doses of cyanocobalamin and hydroxocobalamin (CobaSorb test). In the low status group, the test was performed using a cross-over design with identical doses of both cobalamin forms (1.5, 3, and 6 µg, respectively). In the normal status group, the test was performed with either 3, 6, and 9 µg cyanocobalamin (*n* = 28), or with 9 µg cyanocobalamin and 9 µg hydroxocobalamin (*n* = 14).

**Results:**

In both groups, median ΔholoTC (pmol/L) was higher after intake of cyanocobalamin compared to (hydroxocobalamin) [low status: 1.5 µg: 19 (6); 3 µg: 23 (7); 6 µg: 30 (14); normal status: 9 µg: 30 (13) pmol/L]. Independent of B12 form, no difference was observed in ΔholoTC between those receiving 1.5 and 3 µg in the low status group or 6 and 9 µg cyanocobalamin in the normal status group. However, in both groups, administration of 6 µg cobalamin resulted in a significant higher ΔholoTC than did 3 µg [low status: *p* = 0.02 (0.009) for cyanocobalamin (hydroxocobalamin); normal status: *p* = 0.03 for cyanocobalamin].

**Conclusions:**

Administration of cyanocobalamin resulted in a more than twofold increase in holoTC in comparison with hydroxocobalamin. The absorptive capacity was reached only by doses above 3 µg cobalamin. Our results underscore the importance of using the same form of cobalamin when comparing uptake under different conditions.

**Clinical trial registry number:**

NCT02832726 at https://clinicaltrials.gov and 2016/09/012147 at Clinical Trials Registry India.

**Electronic supplementary material:**

The online version of this article (doi:10.1007/s00394-017-1553-5) contains supplementary material, which is available to authorized users.

## Introduction

Cobalamin (vitamin B12) is an essential micronutrient. Inadequate intake or impaired intestinal absorption leads to cobalamin deficiency and clinical signs of neurological impairment and/or anemia [1].

Cyanocobalamin (CN-Cbl) is the synthetic form of the vitamin, most often used in vitamin pills. In food items, cobalamin is present as hydroxocobalamin (HO-Cbl), or the coenzymes methylcobalamin or 5′-deoxyadenosylcobalamin [2]. Upon light exposure, the coenzymes are converted to HO-Cbl.

A number of studies have investigated the absorption of CN-Cbl, while only a few over 40-year-old studies have compared the uptake of various other forms of the vitamin. Employing radioactive-labeled cobalamin and whole-body monitoring, Weisberg and Glass [3] found that CN-Cbl and HO-Cbl were equally absorbed at large pharmacological dosages (100–1000 µg), and Heinrich and Gabbe [4] and Adams et al. [5] confirmed these findings for low doses of cobalamin (< 5 µg). In more recent time, usage of radioactive-labeled cobalamin has not been considered suitable for human studies. To circumvent this problem, we designed a test that we named CobaSorb [6–8]. In its original design, serum holotranscobalamin (holoTC, active cobalamin) was measured before and after oral intake of three doses of 9 µg CN-Cbl for 2 days [6–8]. Further studies have shown that the test is suitable for judging cobalamin absorption even in a population with a low cobalamin status and that doses as low as 2 µg can be used [9].

The studies performed so far leave three questions unanswered. Does intake of cobalamin present in a vitamin pill (CN-Cbl) and present in food (HO-Cbl) result in similar increase in plasma holoTC? Which physiological dose of cobalamin should be administered to give the highest increase in holoTC? Moreover, is there any difference in the cobalamin-induced increase in holoTC between individuals with a low and a normal cobalamin status? In the present study, we address these questions.

## Subjects and methods

### Participants with low cobalamin status (group A)

Participants were recruited from Pune, India, and the study was carried out at Deenanath Mangeshkar Hospital and Research Center, Pune, India, in the fall of 2015. In total, 62 healthy lacto-vegetarian Indian individuals aged ≥ 18 years were included. Most of the participants were staff at the hospital in Pune, India. Exclusion criteria were use of vitamin pills containing > 1 µg cobalamin within the last 2 weeks, cobalamin injections given within the last year, and any known chronic systemic disease. The participants in group A were divided into six subgroups with 10 or 11 participants in each. Each subgroup underwent the CobaSorb test twice with the same dose (1.5, 3, or 6 µg) of CN-Cbl and HO-Cbl in a cross-over design, with 2 week interval between tests. The study was performed within the confines of the Helsinki Declaration II, and the study was approved by the Institutional Ethics Committee of Deenanath Mangeshkar Hospital and Research Center (Project no. 2015_APR_SN_167). All individuals gave their informed consent before inclusion in the study.

### Participants with normal cobalamin status (group B)

Participants were recruited by advertisements at Aarhus University Hospital and Aarhus University in Denmark in the spring of 2014, and the study was carried out at Aarhus University Hospital, Aarhus, Denmark, during the same period. In total, 45 healthy omnivorous Danish individuals aged ≥ 18 years were included in the study. Most of them were staff at the hospital in Aarhus, Denmark. Exclusion criteria were the same as for group A. However, the number was further adjusted for expected dropouts due to the nature of the longitudinal design. Thirty participants in group B underwent the CobaSorb tests three times with doses of 3, 6, and 9 µg CN-Cbl. Fifteen participants in group B underwent the test twice; once with 9 µg CN-Cbl and once with 9 µg HO-Cbl. The study was performed within the confines of the Helsinki Declaration II, and the study was approved by the Central Denmark Region Ethics Committee (Project no. 1-16-02-484-13). All individuals gave their informed consent before inclusion in the study.

### Study design

**Fig. 1:**
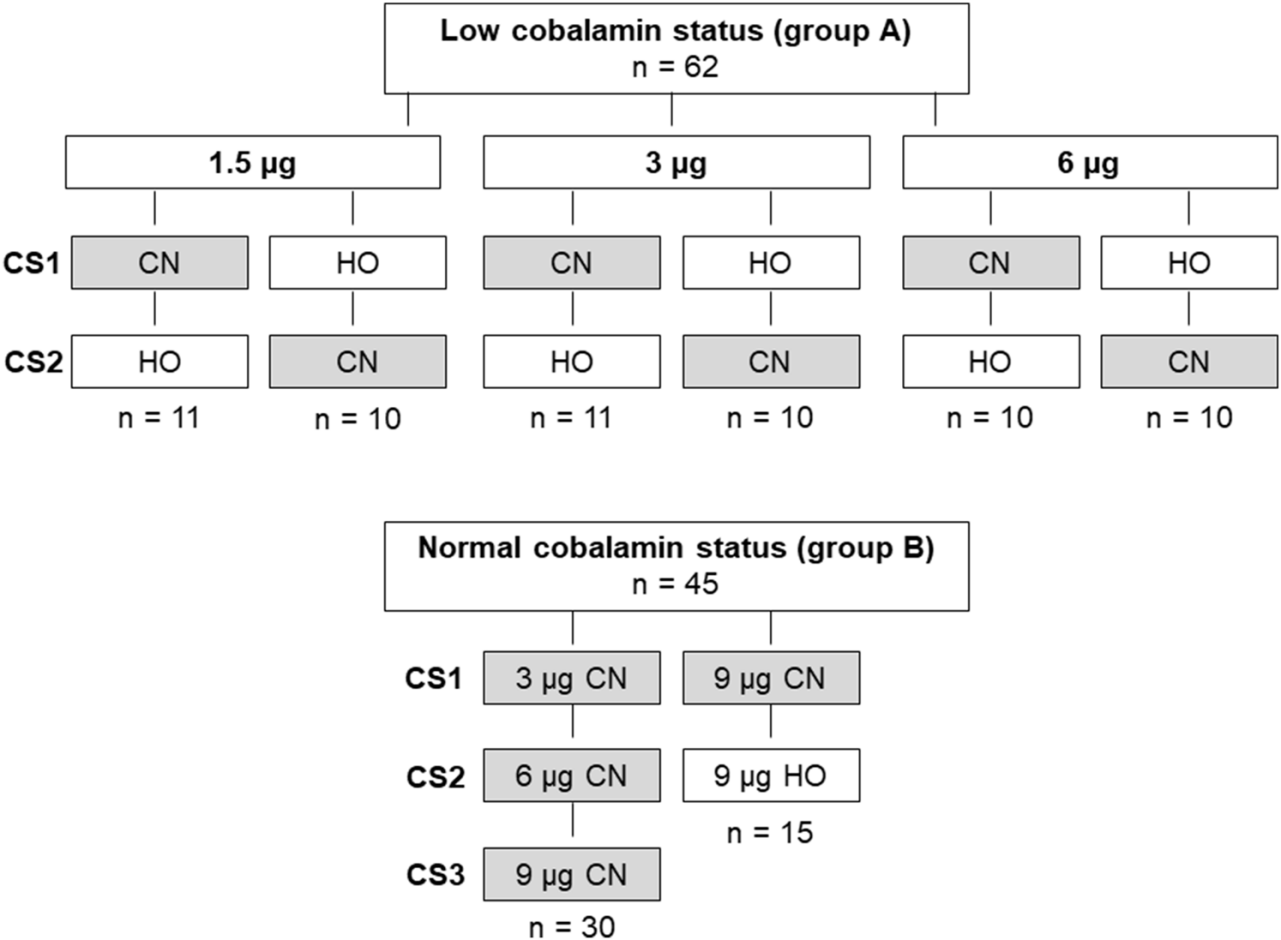
Overview of study design. In the population with low cobalamin status (group A), 62 participants were divided into six groups receiving the same dose (1.5, 3, or 6 µg) of CN-Cbl (CN, grey) and HO-Cbl (HO, white) in a blinded cross-over design. In the population with normal cobalamin status (group B), 30 participants received three doses of CN-Cbl (3, 6, and 9 µg), and 15 participants received the same dose (9 µg) of CN-Cbl and HO-Cbl. The order of CobaSorb tests is shown (first test: CS1; second test: CS2; third test: CS3). There was a wash-out period of 2 weeks between tests to ensure that the holoTC concentrations from the first test had reached baseline concentrations before the next test was carried out. The figure is made in Microsoft Office Powerpoint 2007

We used the CobaSorb test design (see below for details), employing various doses and forms of free (protein unbound) cobalamin (see below for preparation of cobalamin), as indicated in Fig. 1. In a cross-over design, group A (low cobalamin status) received HO-Cbl and CN-Cbl administered in doses of 1.5 µg (*n* = 21), 3 µg (*n* = 21) or 6 µg (*n* = 20) of cobalamin, with an interval of 2 weeks between each test. This strategy was based on earlier findings showing no significant difference in holoTC concentration between baseline and 6 day post-intake of three oral doses of 9 µg CN-Cbl for 2 days [8]. In group B, *n* = 28 received three doses of CN-Cbl (3, 6, and 9 µg) with intervals of 2 weeks between each test, and *n* = 14 received 9 µg of CN-Cbl and 9 µg HO-Cbl, also with 2 week intervals between each dose. All participants were asked to eat their usual diet throughout the study. Most of the Indian participants (group A) reported to be lacto-vegetarian (80%). They all confirmed that they did not have any non-vegetarian meals during the study or 1 week prior to enrollment. The Danish participants (group B) were all omnivorous.

### Preparation of CN-Cbl and HO-Cbl for oral administration

It should be noted that the term HO-Cbl is used to cover both hydroxocobalamin and aquocobalamin. The two forms are interchangeable, and their presence depends on the pH of the solution. For both studies, free cobalamin was given as a liquid solution absorbed in 30 mg of sugar and packed in two-piece hard-shell gelatin capsules (Natur-Drogeriet, Horning, Denmark). CN-Cbl (Betolvex, Actavis, Gentofte, Denmark) and HO-Cbl (Vibeden, Sandox, Copenhagen, Denmark) were dissolved in sterile water, followed by centrifugation for 5 min at 12,000×*g* at room temperature before retaining the supernatant. Concentrations were adjusted to 0.5 mg/ml in sterile water. To ensure that the cobalamin concentrations were accurate, six aliquots of each cobalamin stock were diluted 1:10 in sterile water and converted to diCN-Cbl by incubation with 0.2 M KCN for 1 h in the dark. The absorption of diCN-Cbl at 368 nm was determined using the Shimadzu UV-1800 spectrophotometer (Holm & Halby, Broendby, Denmark), and used to calculate the cobalamin concentration employing a molar absorption coefficient of diCN-Cbl (30,400) [10]. The mean of the six aliquots was used to calculate the stock volume needed to make capsules of 1.5, 3, 6, and 9 µg CN/HO-Cbl.

The cobalamin-sugar capsules were stored in the dark at 4 °C until usage, with a maximal storage time of 6 weeks. To ensure that CN-Cbl (gamma-peak at 361 nm) and HO-Cbl (gamma-peak at 350 nm (aquocobalamin) [10]) remained stable when absorbed in sugar and stored, absorption spectra from 200 to 600 nm were determined with and without sugar, and before and after storage for 6 weeks. No changes occurred during storage (data not shown).

### CobaSorb test

On the morning of day 1, non-fasting blood samples (serum-separating gel tubes) were taken for measurement of baseline holoTC, and the first cobalamin capsule was administered together with water and a snack (fruit or bread). The participants were instructed to take the next capsules after 6 and 12 h, likewise together with a drink and a snack; and to do the same for day 2. On the morning of day 3, non-fasting blood samples (serum-separating gel tubes) were taken for measurement of holoTC.

The blood samples were centrifuged (10 min at 2300×*g*) within 2 h of being drawn, and serum was stored at − 20 °C for later analysis of holoTC. In addition, we collected serum samples for measurement of cobalamin biomarkers and EDTA blood (baseline) for measurement of hematological parameters (see “Biochemical measurements”).

### Biochemical measurements

Serum aliquots from Indian participants were shipped to Denmark on ice for analysis of cobalamin, holoTC, total transcobalamin (totalTC), total haptocorrin (totalHC), and MMA. The samples were frozen upon arrival. Hcy and hematological parameters (see below) were analyzed on EDTA plasma in India. All measures on samples from Danish subjects were analyzed in Denmark.

For each variable, all samples from the same person were measured in one run. HoloTC was measured using an in-house sandwich ELISA for transcobalamin (TC). Prior to analysis, unsaturated transcobalamin (apoTC) was captured by cobalamin-coated magnetic beads [11]. The total imprecision was 8% [11] and the intra-assay imprecision was 4% [12]. The mean holoTC values for the low, intermediate, and high controls were 40, 70, and 114 pmol/L, respectively [12]. The in-house TC ELISA is routinely employed at our department, and had earlier proven to agree with the Active B12 (holoTC) EIA kit (Axis-Shield Diagnostics, Dundee, Scotland, UK) that is available on the AxSYM platform from Abbott [13]. The samples were also measured for totalTC using the above-mentioned TC ELISA. Total HC was measured by an in-house HC ELISA with a total imprecision of 5% and the intra-assay imprecision of 2% [14].

Serum cobalamin, methylmalonic acid (MMA), plasma total homocysteine (tHcy), plasma creatinine, blood hemoglobin (Hb), and erythrocyte mean volume (MCV) were measured on baseline samples. Plasma cobalamin was measured on the Advia Centaur CP Immunoassay System (Siemens). MMA was measured by Liquid Chromatography–Tandem Mass spectrometry on the AB SCIEX Triple Quad 5500 System (AB SCIEX), and tHcy was measured on the Architect Immunoassay Analyzer (Abbott). Hb and MCV were measured on the XN 3000 Hematology Analyzer (Sysmex), and plasma creatinine was measured on the RX Imola (Randox Laboratories) employing routine ISO certified assays.

### Statistics

As this study is the first of its kind using the CobaSorb test with HO-Cbl, we could not perform sample size calculations based on knowledge of assay variation. Instead, we performed post-hoc power calculations by comparing the ΔholoTC mean ± SD of the CobaSorb test with 1.5 µg CN-Cbl (21.6 ± 14.21 pmol/L) and 1.5 µg HO-Cbl (4.95 ± 5.61 pmol/L), the lowest dose used in group A, together with an *α* of 0.05 to justify the sample size and achieved a statistical power of 99.8%.

The D’Agostino–Pearson omnibus test was used to test if data followed the Gaussian distribution. The two-tailed paired *t* test (normally distributed data) or the Wilcoxon signed rank test (not normally distributed data) was used to test for differences between days 1 and 3 concentrations and for differences in ΔholoTC between different CobaSorb tests within the same study group. The unpaired *t* test (normally distributed data) or the Mann–Whitney test (not normally distributed data) was used to compare test results between study groups. Information on the specific statistical tests employed for each set of data is presented in relation to the data presentation and indicated in relevant legends to figures and tables. *p* values < 0.05 were accepted as statistically significant. Data analysis was performed using the statistical software available in GraphPad Prism version 5.

## Results

### Participants

We recruited 62 healthy individuals (32 males and 30 females), with a median [range] age of 32 [18–53] years from the population with low cobalamin status (group A). Of the 62 participants one dropped out, and the data from two others were excluded from the final statistics as outliers. One due to spurious high plasma concentrations of cobalamin [above the upper measurement limit (1467 pmol/L)], and the other due to spurious high plasma levels of holoTC (210 pmol/L) and (totalTC) (2111 pmol/L) {above reference intervals [40–150 (600–1500) pmol/L] [11]}. One participant did not complete the second CobaSorb test, but the data obtained from the first CobaSorb test was included in the final statistical analysis. In the population with normal cobalamin status (group B), we recruited 45 healthy individuals (17 males and 28 females), with a median [range] age of 34 [18–63] years. Three participants dropped out during the study. The baseline values of all participants who completed the study are presented in Table 1.

**Table 1:** Baseline values for the population with low cobalamin status (group A) and the population with normal cobalamin status (group B)

	Ref. Int.	Median [range]		*p* value
		Group A low status *n* = 59 (27 females)	Group B normal status *n* = 42 (27 females)	
Cobalamin (pmol/L)	200–600	110 [61–310]	270 [130–450]	< 0.0001
HoloTC (pmol/L)	40–150	17 [6–120]	69 [32–140]	< 0.0001
TotalTC (pmol/L)	600–1500	960 [600–1800]	710 [510–1160]	< 0.0001
TotalHC (pmol/L)	240–680	710 [360–1370]	610 [300–850]	0.0006
MMA (µmol/L)	0.1–0.3	1.2 [0.14–3.8]	0.21 [0.11–0.48]	< 0.0001
tHcy^a^ (µmol/L) males	6.3–15.7	36 [10–85]	–	–
tHcy^a^ (µmol/L) females	4.9–14.9	19 [8–65]	–	–
Crea (µmol/L) males	60–105	84 [70–110]	83 [60–100]	0.33
Crea (µmol/L) females	45–90	69 [52–90]	68 [53–78]	0.35
Hb (mmol/L) males	8.1–10.3	9.1 [7.4–9.8]	9.6 [8.9–10]	0.002
Hb (mmol/L) females	7.1–9.3	7.5 [5–9]	8.5 [6.9–9.6]	< 0.0001
MCV (fL)	82–98	89 [72–120]	88 [82–96]	0.98

Median with [range] is indicated. Reference intervals are from [11, 14–18]. The unpaired *t* test (normally distributed data) was used to compare group A vs. group B for baseline creatinine and hemoglobin and to compare males vs. females for baseline creatinine and hemoglobin. The Mann–Whitney test (not normally distributed data) was used to compare group A vs. group B for baseline cobalamin, holoTC, totalTC, totalHC, MMA, and MCV; and to compare males vs. females for tHcy in group A. Significant differences (*p* values) between group A and group B are indicated. Statistical significant differences in baseline values between the two populations were found for all biomarkers measured besides creatinine and MCV

Cobalamin, *holoTC* holotranscobalamin, *totalTC* totaltranscobalamin, *totalHC* totalhaptocorrin, *MMA* serum methylmalonic acid, *tHcy* total homocysteine, *crea* plasma creatinine, *MCV* mean cell volume, and *Hb* blood hemoglobin measured at baseline

^a^tHcy was only measured for group A

As expected, group A had an overall low cobalamin status with baseline values of cobalamin and holoTC far below the reference interval (see Table 1). Four subjects did have baseline cobalamin levels above the lower limit of the reference interval (200 pmol/L) [15], but the overall group median (110 pmol/L, *n* = 59) was below the reference interval. This was further supported by overall elevated concentrations of MMA and tHcy, and a combined indicator of cobalamin status (4cB_12_: cobalamin, holoTC, MMA, tHcy) of − 1.7, which indicates possible cobalamin deficiency [19]. However, no indication of macrocytic anemia was present based on values of MCV and Hb. Group B had an overall normal cobalamin status with baseline values of cobalamin-related biomarkers within the reference intervals (Table 1). Eleven subjects did have baseline cobalamin levels just below the lower reference interval limit (200 pmol/L) [15], but the overall group median was 270 pmol/L (*n* = 42). A combined indicator of cobalamin status (3cB_12_: cobalamin, holoTC, MMA) of 0.21 that indicates cobalamin adequacy [19].

Curiously, higher baseline concentrations of the cobalamin-binding proteins, totalTC and totalHC, were found in group A with low cobalamin status, compared with group B with normal cobalamin status (Table 1).

### CobaSorb results

**Fig. 2:**
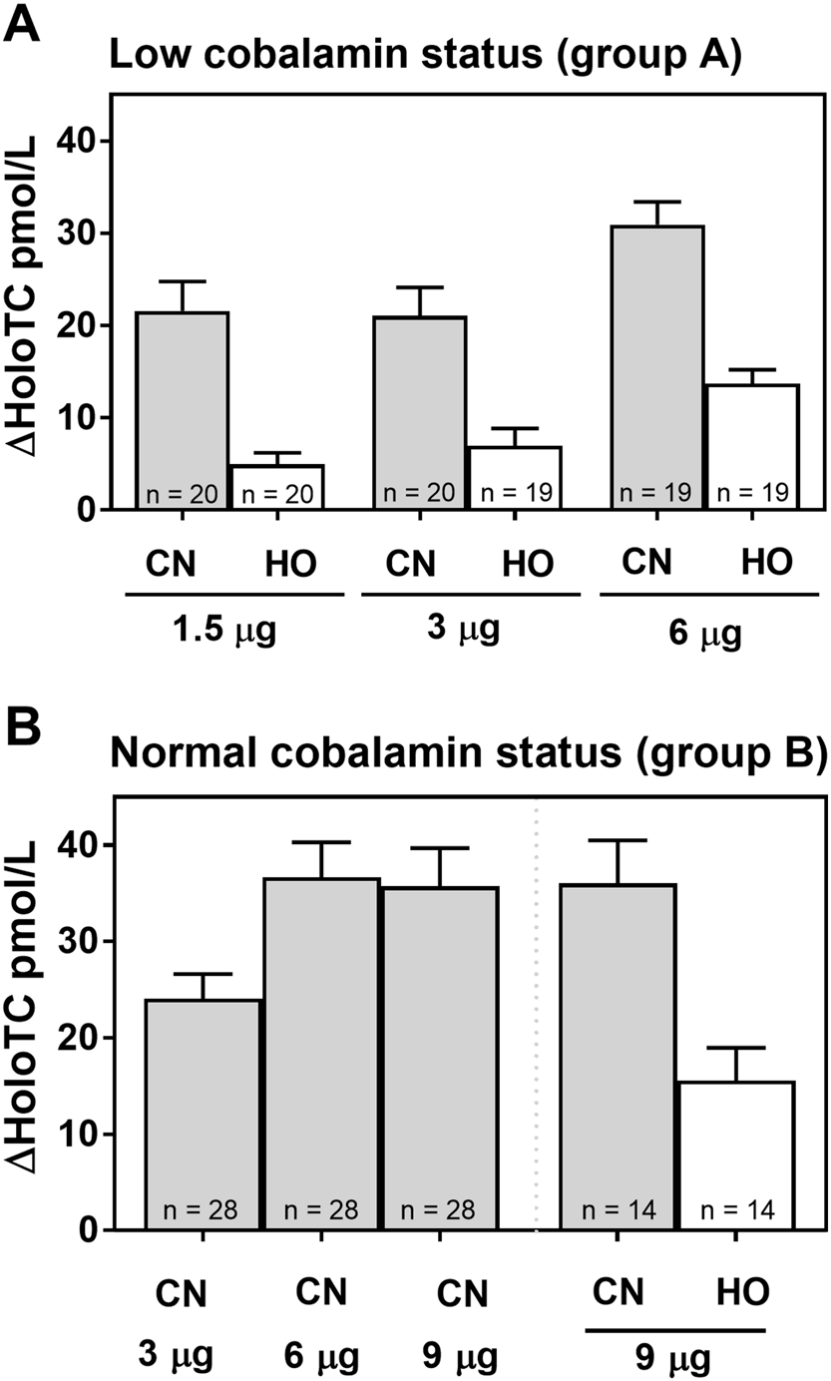
Increase in holoTC upon administration of CN-Cbl and HO-Cbl in the CobaSorb test design. Changes in holoTC (ΔHoloTC, pmol/L) between baseline values and concentrations obtained after intake of three cobalamin capsules for 2 days in a population with low cobalamin status (**a**) (*n* = 59) and a population with normal cobalamin status (**b**) (*n* = 42). Mean values with SEM are presented. The forms [CN-Cbl (CN, grey) and HO-Cbl (HO, white)] and doses (1.5, 3, 6, and 9 µg) of cobalamin are indicated. The increase in holoTC for CN-Cbl was 2–3 times higher than for HO-Cbl in both populations and for all doses tested. In group A, no differences in ΔHoloTC were found between doses of 1.5 and 3 µg for either form of cobalamin. However, doses of 6 µg CN-Cbl showed a higher increase in holoTC than doses of 1.5 µg (*p* = 0.03) and 3 µg (*p* = 0.02), and so did doses of 6 µg HO-Cbl (1.5 µg: *p* = 0.0002; 3 µg: *p* = 0.009). In group B, doses of 6 and 9 µg CN-Cbl showed a higher increase in holoTC than doses of 3 µg (6 µg: *p* = 0.03; 9 µg: *p* = 0.05; paired *t* test); with no difference between doses of 6 and 9 µg. There was no difference in the increase in holoTC between the low cobalamin and normal cobalamin populations for doses of 3 and 6 µg. The figure is made in GraphPad Prism version 5. For details on median [range] increase in holoTC from days 1 to 3 in the CobaSorb, see Table 2

We used a cross-over design (see Fig. 1) for the study performed on group A (low cobalamin status). No difference in baseline holoTC and totalTC was found when CN-Cbl was administered in the 1st test and HO-Cbl in the second test, and vice versa (see Electronic supplementary material Fig. 1). For this reason, we conclude that the order of CobaSorb tests have no influence on the outcome in this study setup, and present the merged results of the CobaSorb tests in Fig. 2 (mean with SEM) and Table 2 (median with range).

**Table 2:** Changes in holoTC (ΔholoTC, pmol/L) during the CobaSorb test in populations with low (group A, *n* = 59) and normal (group B, *n* = 42) cobalamin status

	*n*	Baseline holoTC pmol/L day 1	Post loading holoTC pmol/L day 3	ΔHoloTC increase pmol/L	HoloTC_3_/HoloTC_1_ ratio	*p* value CN vs. HO
Group A						
1.5 µg CN-Cbl	20	23 [10–120]	39 [18–172]	19 [3–53]	1.6	0.0001
1.5 µg HO-Cbl	20	23 [9–109]	26 [12–116]	6 [(− 7)–22]	1.2	
3 µg CN-Cbl	20	17 [7–60]	39 [9–91]	23 [0–55]	2.2	0.0002
3 µg HO-Cbl	19	17 [6–69]	21 [10–82]	7 [(− 10)–29]	1.5	
6 µg CN-Cbl	19	14 [8–91]	48 [18–136]	30 [8–48]	3.4	0.0001
6 µg HO-Cbl	19	15 [7–68]	29 [12–72]	14 [0–24]	2.1	
Group B						
3 µg CN-Cbl	28	75 [32–135]	103 [53–179]	24 [(− 6)–49]	1.4	
6 µg CN-Cbl	28	71 [30–124]	106 [57–195]	32 [8–106]	1.5	–
9 µg CN-Cbl	28	65 [33–133]	105 [53–212]	30 [(− 1)–84]	1.5	–
9 µg CN-Cbl	14	63 [32–144]	100 [59–207]	34 [2–65]	1.6	0.0001
9 µg HO-Cbl	14	73 [29–130]	79 [41–162]	13 [(− 3)–41]	1.2	

Blood samples were removed at baseline (day 1) and after 2 days of loading with 1.5, 3, 6 or 9 µg of CN-Cbl or HO-Cbl (day 3) (see "Subjects and methods" section for details). Median and [range] is indicated, and so is the holoTC (day 3)/holoTC (day 1) ratios. The paired *t* test (normally distributed data) was used in group B to compare ΔholoTC for 9 µg CN-Cbl vs. 9 µg HO-Cbl. The unpaired *t* test (normally distributed data) was used to compare ΔholoTC for 3 µg CN-Cbl vs. 3 µg HO-Cbl and for 6 µg CN-Cbl vs. 6 µg HO-Cbl for group A. The Mann–Whitney test (not normally distributed data) was used to compare ΔholoTC for 1.5 µg CN-Cbl vs. 1.5 µg HO-Cbl for group A. Significant differences (*p* values) between ΔholoTC for CobaSorb tests carried out with the same dose of CN-Cbl and HO-Cbl are indicated. The results (in mean with SEM) are also illustrated in Fig. 2

The CobaSorb results from group A are shown in Fig. 2a and Table 2. The increase in holoTC from days 1 to 3 for doses of 6 µg CN-Cbl [median (range): 30 (8–48) pmol/L] was higher than for doses of 1.5 µg [19 (3–53) pmol/L] (*p* = 0.03) and 3 µg [23 (0–55) pmol/L] (*p* = 0.02). No difference in the increase in holoTC concentration was found between 1.5 and 3 µg CN-Cbl (*p* = 0.1). The same pattern was observed for doses of HO-Cbl. The increase in holoTC for doses of 6 µg HO-Cbl [14 (0–24) pmol/L] was higher than for 1.5 µg {6 [(− 7)–22] pmol/L} (*p* = 0.0002) and 3 µg {7 [(− 10)–29] pmol/L} (*p* = 0.009), and no difference was found between 1.5 and 3 µg HO-Cbl (*p* = 0.34). CN-Cbl showed a 2–3 times higher increase in holoTC concentration than HO-Cbl for all three doses (Fig. 2a, Table 2). No change in totalTC concentration was observed between days 1 and 3 for any form or dose of cobalamin in group A.

The CobaSorb results of group B (normal cobalamin status) with doses of 3 , 6, and 9 µg CN-Cbl and 9 µg HO-Cbl are shown in Fig. 2b and Table 2. The increase in holoTC for 3 µg CN-Cbl {24 [(− 6)–49] pmol/L} was lower than for 6 µg [32 (8–106) pmol/L] (*p* = 0.03) and 9 µg {30 [(− 1)–84] pmol/L} (*p* = 0.005). No difference in the increase in holoTC was found between 6 and 9 µg CN-Cbl (*p* = 0.89). When comparing the increase in holoTC for doses of 9 µg CN-Cbl (34 [2–65] pmol/L) and 9 µg HO-Cbl {13 [(− 3)–41] pmol/L}, the increase in holoTC was 2.8 times higher for CN-Cbl than for HO-Cbl (*p* < 0.0001) (Fig. 2b, Table 2); similar to the findings in group A with low cobalamin status. As for group A, no difference in baseline holoTC concentration was found between subsequent CobaSorb tests in group B (data not shown).

TotalTC showed a median [range] decrease from days 1 to 3 for doses of 9 µg CN-Cbl {(−)53 [(−)145–70] pmol/L (*p* = 0.006)}, but not for doses of 9 µg HO-Cbl {13 [(−)120–85] pmol/L (*p* = 0.92)} in group B.

When comparing the results of the CobaSorb tests for group A (low cobalamin status) and group B (normal cobalamin status), no difference in holoTC increase was observed between the two study populations (3 µg CN-Cbl: *p* = 0.7; 6 µg CN-Cbl: *p* = 0.5). In absolute values, median [range] ΔholoTC was 23 [0–55] pmol/L (group A) and 24 [(−)6–49] pmol/L (group B) for doses of 3 µg, and 30 [8–48] pmol/L (group A) and 32 [8–106] pmol/L (group B) for doses of 6 µg (Table 2).

A few participants in both groups (group A, *n* = 5, group B, *n* = 3) showed no increase or a negative change in holoTC from days 1 to 3 in the CobaSorb test.

## Discussion

We report data on the increase in serum holoTC following administration of various doses of CN-Cbl and HO-Cbl in populations of young healthy individuals with low and normal cobalamin status. Our study has some limitations. It was performed in two ethnically different populations, an Indian (low cobalamin status) and a Danish (normal cobalamin status) population; thus ethnic differences may well be present. In addition, the test used to study uptake of cobalamin, the CobaSorb test, is not able to detect minor increases in holoTC, notably if baseline holoTC is above 65 pmol/L [8]. However, due to the fact that we studied uptake of both forms of cobalamins in various doses in the same individuals, we do not believe that the limitations have major impact on the key conclusions of our studies.

We report that oral administration of CN-Cbl causes a 2–3 times higher increase in holoTC concentration compared with HO-Cbl, independent of test dose and cobalamin status. Initially, we interpreted the result to indicate a better intestinal uptake of free CN-Cbl as compared to HO-Cbl. It is well established that CN-Cbl is more stable than HO-Cbl [20, 21], and thus, it would be reasonable to speculate that HO-Cbl is partially degraded in the rough conditions of the gastrointestinal tract. However, this interpretation conflicts with studies from the 60s and 70s using free radiolabelled cobalamin combined with whole-body counting. These studies suggest an equally efficient intestinal absorption rate of CN-Cbl and HO-Cbl in humans [3–5]. In addition, older studies in rats and also our recent rat study confirm an equal absorption of labeled CN-Cbl and HO-Cbl as judged from monitoring of the total absorption [4, 22]; a result in accord with our present findings in humans. These findings cannot be further interpreted based on our study in humans, but the rat studies allow us to suggest an explanation model. In the rat studies, HO-Cbl is taken up by the liver much more efficiently than is CN-Cbl [4, 22]. Taken together, the results in both human and rat suggest that the intestinal uptake of free CN-Cbl and HO-Cbl are alike, but that the circulating HO-Cbl is absorbed faster by the tissues than CN-Cbl, thereby resulting in a higher circulating level of holoTC after administration of CN-Cbl than that of HO-Cbl. This in turn questions whether oral HO-Cbl is superior compared to CN-Cbl for ensuring an optimal cobalamin status. Obviously, this question cannot be answered by the current study or by animal studies. However, our results point to a limitation in using plasma levels to judge absorption of cobalamin. For example, if the aim is to compare uptake of free cobalamin with food cobalamin from measures of an increase in circulating cobalamin, it is essential that the free and the food-bound cobalamin is administered in the same molecular form. In the past, several studies have suggested that food-bound cobalamin is absorbed less efficiently than free cobalamin [23–25]. Currently, we do not know whether this statement is driven by the fact that most clinical studies use CN-Cbl as free cobalamin, while food cobalamin is HO-Cbl or the coenzyme forms [2]. Based on our data, the absorption of food bound and free cobalamin can only be compared if the same form of cobalamin is present as food bound or free. A possible difference between free CN-B12 and food-bound HO-B12 could be driven by differences in the form of B12 rather than free or food bound.

In our study, we had to consider that a change in holoTC from days 1 to 3 in the CobaSorb test can only be detected if it differs significant from baseline holoTC. For example, if baseline holoTC is low (e.g., 17 pmol/L; median group A) you may see a small increase (e.g., 5 pmol/L) from days 1 to 3. Whereas such a small increase may disappear in the analytical imprecision if baseline holoTC is high (e.g., 69 pmol/L; median group B). Because of this limitation, our choice was to use higher doses of cobalamin in group B (3, 6, and 9 µg) than in group A (1.5, 3, and 6 µg).

In both group A (*n* = 5) and group B (*n* = 3), a few subjects did not respond to the oral supplementation or even showed a small negative change in holoTC from days 1 to 3 in the CobaSorb test. It is known that a high baseline holoTC (> 65 pmol/L) may influence the CobaSorb test outcome [8], and this may have been at play for two of the participants with baseline holoTC of 76 and 78 pmol/L, respectively. However, the other six participants had baseline holoTC within the range of 16–59 pmol/L. They all showed an increase in holoTC in their other CobaSorb tests; thus, it is unlikely that the lack in response is caused by persisting malabsorption.

The dose of 3 and 6 µg CN-Cbl was administered to both groups and the response to these doses could, therefore, be compared. The two groups differed in cobalamin status due to diet preferences (lacto-vegetarian vs. omnivorous), but was comparable from the point of view that both were healthy young individuals—mostly hospital employees. Interestingly, we observed no difference in the induced increase of holoTC concentrations between the groups. We interpret this to indicate a comparable absorption of CN-Cbl independent of cobalamin status. For both groups, we found a dose-dependent increase in holoTC, and based on the combined results, we conclude that the maximal absorption is reached with a dose between 3 and 6 µg of free cobalamin three times per day, respectively. This conclusion contradicts with the general belief that the maximal absorptive capacity of the intestinal intrinsic factor receptor is met with 1–2 µg of cobalamin per meal [26, 27].

Curiously, higher baseline concentrations of totalTC and totalHC were found in group A with low cobalamin status compared with group B with normal cobalamin status. Earlier studies have shown that totalTC and totalHC are unaffected by cobalamin status [28]. We speculate if our reported differences are of ethnical origin.

In conclusion, we have shown that oral intake of free CN-Cbl induced a higher increase in circulating holoTC concentrations than free HO-Cbl and that both individuals with a low and a normal cobalamin status have an absorptive capacity that is saturated above doses of 3 µg three times a day. Further studies are needed to determine if CN-Cbl or HO-Cbl provides the best oral source for ensuring a sufficient tissue supply of cobalamin.

## Electronic supplementary material

Below is the link to the electronic supplementary material.

Supplementary material 1 (DOCX 217 KB)

## Abbreviations

ApoTC: Unsaturated transcobalamin
CN-Cbl: Cyanocobalamin
Hb: Blood hemoglobin
HO-Cbl: Hydroxoaquocobalamin
HoloTC: Holotranscobalamin (active cobalamin)
MCV: Mean cell volume
MMA: Methylmalonic acid
tHcy: Plasma total homocysteine
TotalHC: Total haptocorrin
TotalTC: Total transcobalamin
