# Supplementary material for: Increase in circulating holotranscobalamin after oral administration of cyanocobalamin or hydroxocobalamin in healthy adults with low and normal cobalamin status
Source: Eur J Nutr. 2017 Oct 16;57(8):2847–55. doi: 10.1007/s00394-017-1553-5 (PMC6267412; doi:10.1007/s00394-017-1553-5)
Supplement: Supplementary file 1 — Supplementary material 1 (DOCX 217 KB) [file 394_2017_1553_MOESM1_ESM.docx]

**Electronic supplementary material Fig 1** Baseline holoTC (A) and totalTC (B) measures from subsequent CobaSorb tests carried out in a population with low cobalamin status (group A). The form of cobalamin (cyanocobalamin (CN, grey) or hydroxocobalamin (HO, white)) is indicated and so is the test order (1^st^ test: CS1 or 2^nd^ test: CS2). Medians are shown, and differences between subsequent tests are determined by the Wilcoxon signed rank test. No difference in baseline holoTC (A) or totalTC (B) was observed between subsequent tests with the exception of baseline totalTC for group 5. The high baseline holoTC observed in group 1 is caused by a single outlier with a baseline holoTC of 120 pmol/L. The figure was made in Graph Pad Prism version 5. Abbreviations: holoTC: holotranscobalamin; totalTC: totaltranscobalamin
